# Inducible Ablation of Melanopsin-Expressing Retinal Ganglion Cells Reveals Their Central Role in Non-Image Forming Visual Responses

**DOI:** 10.1371/journal.pone.0002451

**Published:** 2008-06-11

**Authors:** Megumi Hatori, Hiep Le, Christopher Vollmers, Sheena Racheal Keding, Nobushige Tanaka, Christian Schmedt, Timothy Jegla, Satchidananda Panda

**Affiliations:** 1 The Salk Institute for Biological Studies, La Jolla, California, United States of America; 2 Genomics Institute of Novartis Research Foundation, San Diego, California, United States of America; 3 Department of Cell Biology and Institute for Childhood and Neglected Diseases, The Scripps Research Institute, La Jolla, California, United States of America; Temasek Life Sciences Laboratory, Singapore

## Abstract

Rod/cone photoreceptors of the outer retina and the *melanopsin*-expressing retinal ganglion cells (mRGCs) of the inner retina mediate non-image forming visual responses including entrainment of the circadian clock to the ambient light, the pupillary light reflex (PLR), and light modulation of activity. Targeted deletion of the *melanopsin* gene attenuates these adaptive responses with no apparent change in the development and morphology of the mRGCs. Comprehensive identification of mRGCs and knowledge of their specific roles in image-forming and non-image forming photoresponses are currently lacking. We used a Cre-dependent GFP expression strategy in mice to genetically label the mRGCs. This revealed that only a subset of mRGCs express enough immunocytochemically detectable levels of melanopsin. We also used a Cre-inducible diphtheria toxin receptor (iDTR) expression approach to express the DTR in mRGCs. mRGCs develop normally, but can be acutely ablated upon diphtheria toxin administration. The mRGC-ablated mice exhibited normal outer retinal function. However, they completely lacked non-image forming visual responses such as circadian photoentrainment, light modulation of activity, and PLR. These results point to the mRGCs as the site of functional integration of the rod/cone and melanopsin phototransduction pathways and as the primary anatomical site for the divergence of image-forming and non-image forming photoresponses in mammals.

## Introduction

**Figure 1:**
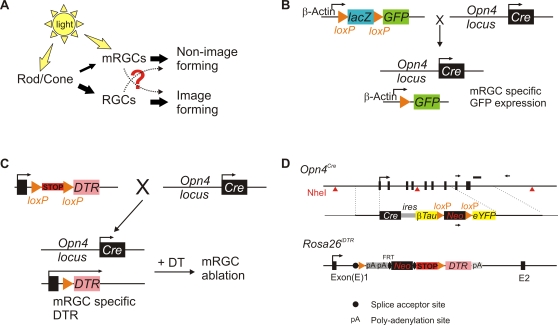
Strategy for fluorescent labeling or inducible ablation of mRGC lineage in the mouse retina. (A) The cellular circuitry underlying the rod/cone and mRGC contribution to visual responses. Thickness of the filled arrows roughly highlights the relative strength of information flow. (B) Strategy to fluorescently label mRGCs by breeding a mouse carrying a *Cre* recombinase “knocked-in” to the *melanopsin* promoter to *Z/EG* mouse that allows Cre-dependent expression of GFP from chicken beta-actin promoter. (C) Strategy to achieve inducible and specific ablation of mRGCs. *Opn4^Cre/+^* mouse was bred to a mouse expressing *Cre*-dependent expression of simian diphtheria toxin receptor. The resulting progeny develop normal mRGCs expressing DTR, which allows specific ablation of these cells by DT administration. Schematic of two targeting vectors used to achieve inducible mRGC ablation are shown in (D). The *Cre* knock-in cassette for targeted insertion to *melanopsin* locus also carried coding sequences for CRE dependent expression of βTau-eYFP. However, fluorescence from βTau:eYFP was undetectable in retina from *Opn4^Cre/+^* mice (data not shown). The targeting vector and generation of *R26^iDTR/+^* mice are described in [20]. A schematic of the targeting vector is shown here.

The rod/cone photoreceptors of the outer retina signal via multisynaptic pathways to the retinal ganglion cells (RGCs) of the inner retina. The RGCs, in turn, transmit the visual information to the brain via their axonal projections. A small subset of RGCs exclusively expresses the functional photopigment melanopsin (OPN4) and is intrinsically photosensitive, but also receives rod/cone inputs (Figure 1A) [1]-[5]. These mRGCs, along with the rod/cone photoreceptors, mediate several non-image forming, or adaptive ocular photoresponses (AOPs), which help organisms optimize their physiological performance in variable ambient light conditions. These AOPs include rapid adjustment of pupil size, modulation of general activity and endocrine function, and tuning of the phase and period length of the circadian clock to adapt to the light environment (reviewed in [6]).

Mouse genetics has established the complementary roles of both rod/cone and melanopsin in these AOPs. Mice with outer retinal degeneration (*rd/rd*) retain functional mRGCs and exhibit almost intact AOPs [7]–[11]. However, these adaptive responses are completely abolished in mice that lack both melanopsin and functional outer retina photoreceptors, thus establishing a dominant role of melanopsin photopigment in these processes [12], [13]. Yet, the AOPs are attenuated, not eliminated, in melanopsin deficient (*Opn4^−/−^*) mice, an observation that suggests a role for rod/cone photoreceptors in AOPs [14]–[16]. The mRGCs develop normally in *Opn4^−/−^* mice and make normal monosynaptic projections to the suprachiasmatic nucleus (SCN) and olivary pretectal nucleus (OPN) which regulate the circadian behavior and pupillary constriction, respectively [14]. Both the SCN and OPN also receive direct and indirect projections from RGCs that do not express melanopsin [17]. Therefore, it is unclear whether rod/cone-initiated light signal is transmitted primarily via the mRGCs or via other RGCs to brain regions that regulate AOPs. Additionally, the mRGCs have also been suggested to play a role in modulating rod/cone initiated image-forming functions [18], [19].

To understand the specific role of mRGCs in both image-forming and adaptive ocular photoresponses, we generated mice expressing Cre-inducible diphtheria toxin receptor exclusively in the mRGC lineage. Diphtheria toxin (DT) crosses the blood-brain barrier after systemic injection and has been shown to trigger cell death in neurons expressing a primate DTR without triggering a significant immune response [20], [21]. This strategy allows normal embryonic and postnatal differentiation and development of the target cell type which can be verified in adult mice. Subsequent acute cell ablation with local or systemic DT administration circumvents any potential developmental compensation. Systemic DT injection in the adult mice with DTR expressing mRGCs triggers a profound loss of mRGCs. The mRGC-ablated mice lose non-image forming visual responses while maintaining largely intact image-forming functions. This demonstrates a central role of the mRGCs as the site of integration of melanopsin and rod/cone initiated photoresponses for generating adaptive light responses in mammals.

## Results

### Fluorescent tagging of mRGCs

**Figure 2:**
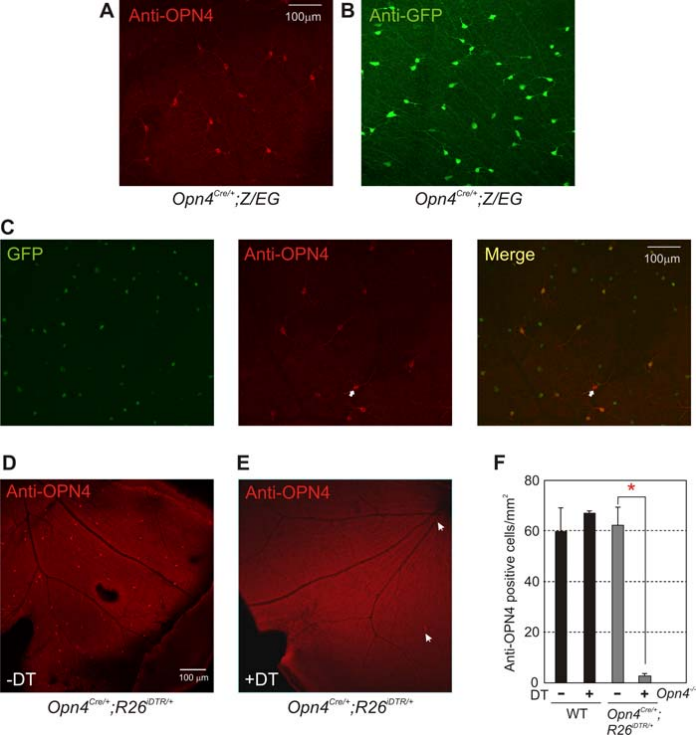
Cre-dependent GFP labeling and inducible ablation of mRGCs. Retina of adult *Opn4^Cre/+^;Z/EG* mouse probed with (A) anti-OPN4 or (B) anti-GFP antibodies show staining of a small subset of cells. (C) Only a subset of GFP positive cells also stained with anti-OPN4 antibody. A section of the flat mount retina containing a cell (marked with an arrow) that stained with melanopsin antibody, but did not express detectable level of GFP is shown. (D) Retina of *Opn4^Cre/+^;R26^iDTR/+^* showed normal melanopsin immunostaining in a small fraction of RGCs. (E) Two weeks after DT administration, the number of melanopsin-immunoreactive cells were significantly reduced. (F) Average melanopsin immunoreactive cell density in WT and *Opn4^Cre/+^;R26^iDTR/+^* retina was comparable. Two weeks after DT administration, the number of cells in WT retina remained unchanged, while that in *Opn4^Cre/+^;R26^iDTR/+^* retina reduced from 62.3 mm^−2^ to 2.8 mm^−2^. No melanopsin immunostaining was observed in *Opn4^−/−^* mice. It is important to note that retina from all DT-treated mice tested by immunostaining still retained a few melanopsin staining cells (arrows), implying incomplete expression of Cre in all mRGCs and/or insufficient level of bioavailable DT. Average cell counts (+SEM, n = 3 to 5 retinas) from each genotype/treatment group are shown. Significant difference in cell numbers (Student's *t* test, *p*<0.05) between mRGCs without and with DT was highlighted with an asterisk.

To comprehensively tag mRGCs with transcriptionally active *melanopsin* locus, we generated a mouse line which carries a Cre recombinase and a Cre-dependent *βTau*-Yellow Fluorescent Protein (YFP) expression cassette knocked-in to the native *melanopsin* locus (Figure 1). However, no detectable YFP expression was found in the retina of *Opn4^Cre/+^* mouse, which may be due to weak transcriptional activity from the native *melanopsin* promoter and/or low expression of the second transcript downstream of an internal ribosomal entry site (IRES) cassette. Next, we bred the *Opn4^Cre/+^* mouse with *Z/EG* mouse (Figure 1B) [22]. This strategy allows Cre-dependent expression of green fluorescent protein (GFP) from a strong *β-actin* promoter, such that GFP is uniformly expressed in all mRGCs irrespective of the heterogeneity in the level of transcription from the native *melanopsin* locus or in the level of immunologically detectable melanopsin protein. In the retina of adult *Opn4^Cre/+^;Z/EG* mice, GFP expressing cells were mostly found in the RGC sub-layer, and these cells had extensive dendritic arborization characteristic of the mRGCs. An average of 131 GFP expressing cells/mm^2^ (±25.4, SD, n = 3) were found in these retina, 42.6% of which also expressed immunologically detectable levels of melanopsin (Figures 2A–2C). These cells with detectable levels of melanopsin protein likely represent the M1 type mRGCs [23]. The second group of GFP positive mRGCs may express very low level of melanopsin representing the M2 type of mRGCs [23], or may represent cells where melanopsin promoter is almost silent in adulthood. A small number of RGCs stained positive for melanopsin, but showed no detectable level of GFP fluorescence. This may represent cells with insufficient Cre expression, Cre activity, or GFP level. In summary, GFP expression pattern in *Opn4^Cre/+^;Z/EG* mice established (a) restricted expression of melanopsin in RGC layer and (b) sufficient CRE activity in vast majority of both M1 and M2 type mRGCs.

### Specific ablation of mRGCs in adult mice

To achieve inducible ablation of mRGC lineage, we bred the *Opn4^Cre/+^* mouse with a mouse strain in which the inducible diphtheria toxin receptor (simian *Hbegf*) (iDTR) is knocked-in to the *ROSA26* locus (*R26^iDTR^*). The iDTR can only be expressed after Cre-mediated excision of a transcriptional STOP cassette [20] (Figures 1C and 1D). As melanopsin function is haplosufficient in mice [14], this approach allows normal differentiation and function of the melanopsin cell lineage in the double heterozygote mice (*Opn4^Cre/+^;R26^iDTR/+^*) and their specific ablation upon DT administration.

**Figure 3:**
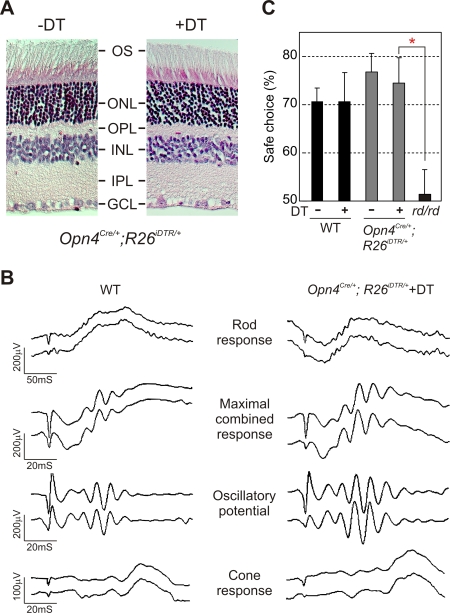
mRGC ablation does not alter the normal retina architecture and image-forming responses. (A) Hematoxylin and Eosin staining of 5 μm thick paraffin embedded sections of retina from *Opn4^Cre/+^;R26^iDTR/+^* mice without and with DT injection. DT application had no detectable adverse effect on the normal stratification of the retina (outer segment (OS), outer nuclear layer (ONL), outer plexiform layer (OPL), inner nuclear layer (INL), inner plexiform layer (IPL), and ganglion cell layer (GCL)). (B) Representative full-field ERG of WT and DT-treated *Opn4^Cre/+^;R26^iDTR/+^* mice showing rod, cone and maximal combined responses. Responses from both eyes were simultaneously measured and plotted. Quantitative analysis of magnitude and timing of a-wave, b-wave and oscillatory potentials of these two genotype groups (3 mice each) showed no significant difference (data not shown). (C) Image forming visual function as assessed by the visual cliff test was unaffected by mRGC ablation. Average percentage (+SEM, n = 5 to 13 mice) of positive choice in 10 trials for each mouse are shown. Mice with outer retina degeneration (*rd/rd*) made random choices while stepping down from the platform and were significantly different (Student's *t* test, *p*<0.05; red asterisk) from the other four groups. No significant difference in test performance was found among native or DT-treated WT or *Opn4^Cre/+^;R26^iDTR/+^* mice.

The retina of 8 week old adult *Opn4^Cre/+^;R26^iDTR/+^* mice showed normal stratification and density of melanopsin-immunostaining (Figures 2D and 3A). Two weeks after DT administration (intraperitoneal injections of DT at 50 μg/kg body weight, 2–3 times at 3 d apart) there was a dramatic reduction of mRGCs by over 90% in *Opn4^Cre/+^;R26^iDTR/+^*(Figure 2E), but not in wild type (WT) mice carrying only one or no copy of either of the two transgenes (Figure 2F). The DT administration did not cause any widespread cell death in the retina as the normal stratification of the major cell layers remained intact (Figure 3A). This demonstrated the accessibility of DT across the blood-retina barrier and the success of the iDTR system in specific ablation of differentiated RGC subtypes in live mice.

### Normal image-forming visual responses in mRGC ablated mice

Image-forming vision requires normal retina architecture, light-induced electrical activities in the retina and RGC-mediated transmission of the light information to the image processing brain centers. As shown in Figure 3A, DT-induced mRGC ablation had minimal impact on the overall retina architecture. These mice also exhibited normal electroretinogram (ERG) (Figure 3B). Rod-mediated responses to scotopic illumination, cone-mediated responses to photopic illumination after photobleaching of rods and maximal rod/cone combined responses were comparable in WT and *Opn4^Cre/+^;R26^iDTR/+^* mice injected with DT. Finally, to evaluate pattern forming visual responses, we tested the performance of mice in a visual cliff test (Figure 3C). The test evaluates the ability of the mice to visually discriminate between a “safe” and an “unsafe” landing space and accordingly step down from a slightly raised platform to the safe area. Typically, mice with intact pattern forming vision make correct decisions and step on the safe side in >70% of trials, while mice lacking image-forming vision make random choices and choose the safe side in ∼50% of trials. Both WT and *Opn4^Cre/+^;R26^iDTR/+^* mice treated with or without DT performed equally and made the safe choice in >70% of 10 repeated trials, while mice with outer retina degeneration (*rd/rd*) made random choices. These results indicate that the mRGCs do not play a major role in the overall image-forming and visual responses in mammals, which is consistent with the limited monosynaptic projections of the mRGCs to image processing brain regions. Nonetheless, mRGCs may still play some roles in visual responses. For instance, the loss of PLR may cause light-induced damage to the retina and thereby affect image-forming responses.

### Loss of PLR in mRGC ablated mice

**Figure 4:**
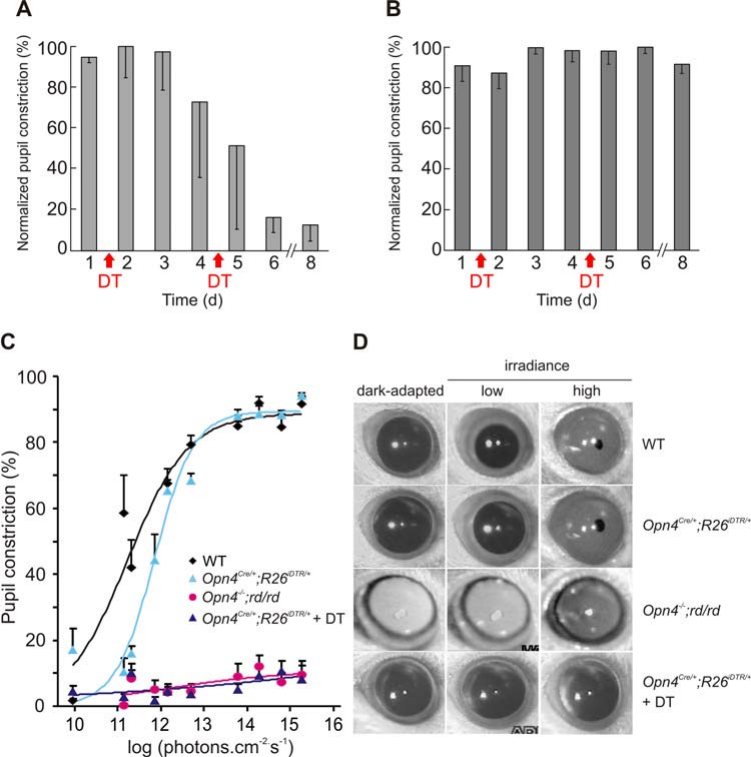
Necessity of mRGCs for PLR. DT injection severely attenuates pupil constriction in response to 20 μW of monochromatic blue light (470 nm) in *Opn4^Cre/+^;R26^iDTR/+^* mice (A), but does not affect in such PLR response in WT mice (B). Normalized pupil constriction (-SD; n = 3 mice) measured one day prior to or every day following DT injection for up to 8 days are shown. There was variability in the rate of loss in PLR response among *Opn4^Cre/+^;R26^iDTR/+^* as reflected in the larger error bars. (C) Pupil constriction in response to varying irradiance levels over 5 log units shows the necessity of mRGC for PLR. Average (+SEM, n = 5 to 6 mice) and fitted sigmoid curves for WT, *Opn4^Cre/+^;R26^iDTR/+^*, *Opn4^−/−^;rd/rd* and DT-treated *Opn4^Cre/+^;R26^iDTR/+^* mice are shown. (D) Representative frozen video images showing dark adapted pupil and pupil under low (10^11^ photons.cm^−2^.s^−1^) or high intensity light (10^15^ photons.cm^−2^.s^−1^) of 470 nm are shown. Notice the complete lack of pupil constriction in *Opn4^−/−^;rd/rd* and in DT-treated *Opn4^Cre/+^;R26^iDTR/+^* mice. For each genotype representative images of the same eye under three different conditions are shown.

Both rod/cone photoreceptors and melanopsin play complementary roles in dynamically adapting the pupil size to ambient light. Specifically, rod/cone photoreceptors mediate response to low light intensity, while melanopsin partly regulates pupil constriction under high intensity light conditions [12]–[14], [24]. The OPN, which mediates PLR, receives a significant number of mRGC projections [4], [17]. To test the role of mRGCs in the non-image forming visual responses, we measured PLR at 20 μW of monochromatic blue light (470 nm, 10 nm half-peak width). This intensity of light triggers ∼80% pupil constriction in WT mice. Both WT and the *Opn4^Cre/+^;R26^iDTR/+^* mice showed comparable constriction to this light intensity (data not shown). After two doses of DT injection (50 μg/kg, 3 d apart), WT mice maintain normal pupil constriction. However, during the course of a week following the first DT injection, PLR in the *Opn4^Cre/+^;R26^iDTR/+^* mice gradually lost sensitivity (Figures 4A and 4B). The rate of loss in PLR was highly variable among this group. Almost 90% sensitivity was lost in the first week, and complete loss of PLR as in *Opn4^−/−^;rd/rd* mice was observed after the second week following DT injection (Figures 4C and 4D). The loss in PLR, coupled with normal ERG in DT-treated *Opn4^Cre/+^;R26^iDTR/+^* mice implies a necessary role of mRGCs in dynamic adaptation of pupil size to ambient light.

### Loss of circadian photoentrainment in mRGC ablated mice

**Figure 5:**
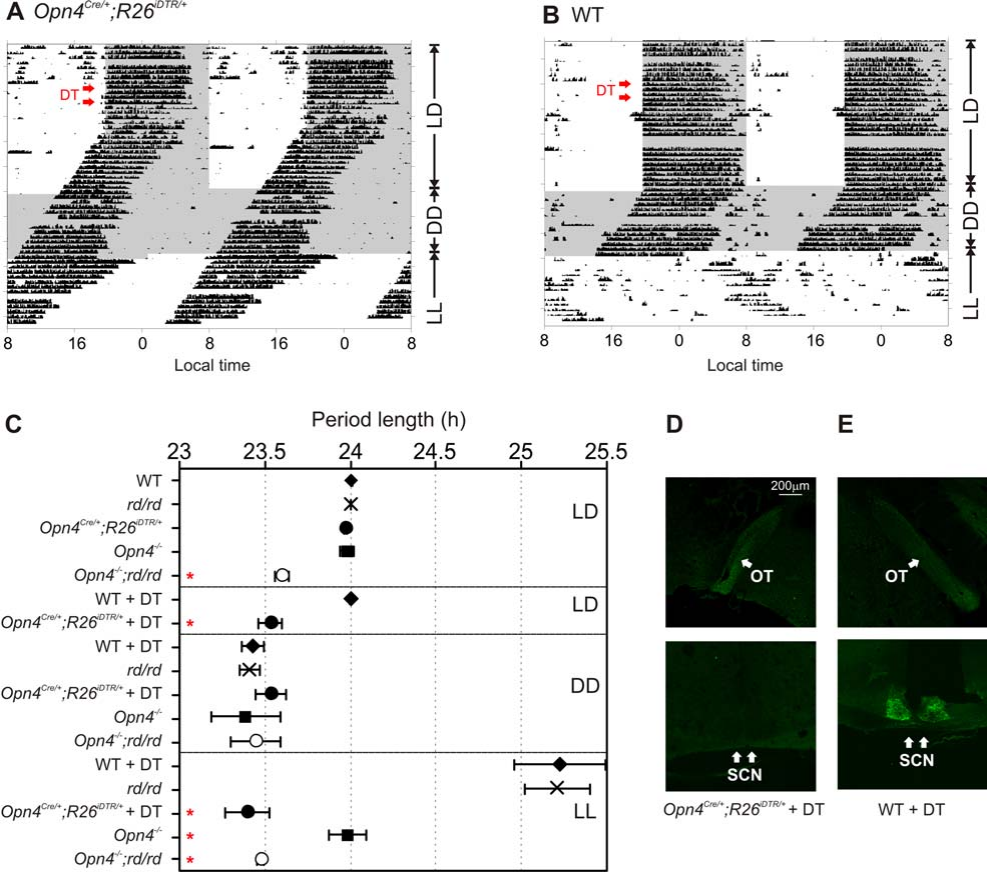
mRGCs are necessary for light adaptation of circadian wheel running activity rhythm. Representative daily wheel running activity profile of an (A) *Opn4^Cre/+^;R26^iDTR/+^* and (B) WT mouse under 12 h light∶12 h dark (LD), constant dark (DD) or constant light (LL) are shown. A week after DT injection (red arrows) the wheel running activity of *Opn4^Cre/+^;R26^iDTR/+^* began to “free run” with a periodicity similar to that under no light. Constant light had no effect on the daily drift in activity onset in this mouse. The wheel running activity of the WT mouse remained entrained to the LD cycle even after DT injection and was lengthened under constant light. Daily wheel running activity profile of mice were binned in 6 min and double plotted such that the activity from consecutive days are plotted to the right and beneath the data from previous day. Periods of darkness are shown in shaded area. (C) Genotypes of mice and their respective average (±SEM, n = 3 to 6 mice) period length of wheel running activity rhythm under conditions of LD, DD and LL as determined by periodogram analysis in Clocklab software [41] are shown. Within each lighting or treatment group (separated by solid box) significant difference (Student's *t* test, *p*<0.05) from WT group is shown by red asterisk. (D) Anterograde CTB-Alexa Fluor 488 tracing in the optic tract (OT) is intact, but is completely abolished in the SCN of DT treated *Opn4^Cre/+^;R26^iDTR/+^* mice. (E) Staining in both regions are left intact in WT mice treated with DT.

We next tested the effect of mRGC ablation on circadian photoentrainment. The circadian wheel running activity of mice has an intrinsic periodicity of less than 24 h. Photic input to the SCN makes daily phase adjustments to the clock so that the animal's activity rhythm maintains a constant phase relation with the ambient photoperiod (reviewed in [25]). The daily wheel running activity of *Opn4^Cre/+^;R26^iDTR/+^* and of WT mice entrained normally to an imposed 12 h light:12 h dark (LD) cycle (Figures 5A and 5B). The mice consolidated their activity with activity commencement juxtaposed to the dark onset. After 10 days of entrainment all mice were administered DT. The WT mice continued to exhibit 24 h rhythm in activity-rest cycle with the activity onset coincident with the dark onset. However, after one week following DT injection, the *Opn4^Cre/+^;R26^iDTR/+^* mice phenocopied the *Opn4^−/−^;rd/rd* mice showing no sign of functional light input to the circadian clock. Their activity rhythm under LD conditions exhibited a period length of ∼23.5 h which is similar to that under constant dark (DD) conditions (Figure 5C).

To clearly establish the roles of retina photopigments and photoreceptor cells in circadian entrainment, we compared the temporal activity rhythms of DT-treated WT and DT-treated *Opn4^Cre/+^;R26^iDTR/+^* with those of *rd/rd*, *Opn4^−/−^* and *Opn4^−/−^;rd/rd* mice under three different lighting conditions: LD, DD and LL (constant light). Mice with complete loss of photopigments or acute loss of mRGC lineage in adulthood showed a DD period length that was indistinguishable from that of WT mice, thus implying normal function of the SCN oscillator sustains without tonic input from the mRGCs. As shown before [8], [15], [16] under LD and LL conditions, the circadian clock in *rd/rd* and in *Opn4^−/−^* mice showed signs of light input. Both genotypes entrained normally to LD cycle; i.e. they exhibited 24 h LD period length. As reported earlier [13], [15], [16], under constant light, both *rd/rd* and *Opn4^−/−^* mice showed period lengthening–although the effect was less pronounced in the latter genotype. A parsimonious interpretation of circadian photoentrainment in melanopsin photopigment deficient (*Opn4^−/−^*) mice, but not in mRGC-ablated (DT-treated *Opn4^Cre/+^;R26^iDTR/+^*) mice is the photic input to the circadian clock in *Opn4^−/−^* mice must initiate from the outer-retina rod/cone photoreceptors and transmit through the mRGCs (which are intact in these mice). Degeneration or dysfunction of the outer retina photoreceptors along with lack of melanopsin photopigment or acute ablation of mRGCs severs light input to the SCN.

At the end of the circadian wheel running experiments, we intravitreally injected Alexa Fluor 488 conjugated Cholera Toxin subunit B (CTB) to the eyes of DT treated WT and *Opn4^Cre/+^;R26^iDTR/+^*mice and monitored staining of the RGC axons and terminals in the brain. Brain sections of both genotypes showed normal staining in the optic tract (OT), which is known to consist of projections from both mRGCs and other RGCs. However, the SCN of *Opn4^Cre/+^;R26^iDTR/+^*mice showed very little staining, while the SCN of WT mice showed significant amount of fluorescence (Figures 5D and 5E). Therefore, the loss of melanopsin immunostaining in the retina along with the loss of a majority of projection to the SCN, but not in the OT of DT treated *Opn4^Cre/+^;R26^iDTR/+^*mice firmly suggests specific ablation of mRGCs in these mice.

### mRGCs mediate light suppression of activity

**Figure 6:**
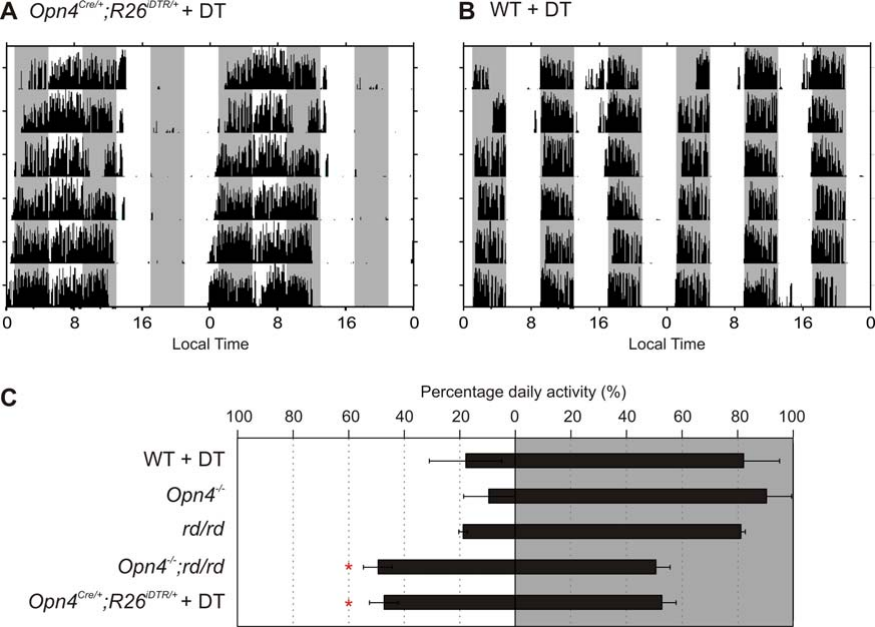
Lack of negative masking in mRGC ablated mice. Representative wheel running activity profile of DT-treated (A) *Opn4^Cre/+^;R26^iDTR/+^* and (B) WT mice held under ultradian cycle of 4 h light and 4 h darkness are shown. The sessions of darkness are shown by shaded area. Activity profile is plotted as in Figure 5A. (C) Average percentage (±SEM, n = 3 to 6 mice) of daily activity during the bouts of light sessions (on left) or during darkness (on right) for five different groups of mice are shown. Groups with percent activity during light phase significantly different (Student's *t* test, *p*<0.05) from that of the DT-treated WT mice are shown with asterisk.

The general locomotor activity of nocturnal rodents is acutely suppressed upon exposure to photopic light levels [26]. Such activity masking by light is left almost intact in *rd/rd* mice [27], mildly attenuated in *Opn4^−/−^* mice [28], and is abolished in *Opn4^−/−^;rd/rd* mice [13]. Multiple brain centers are involved in this response, which requires photic input from the retina. To assess the role of mRGCs in negative masking, we measured the wheel running activity of mice subjected to a short photocycle of 4 h light and 4 h dark over several days (Figure 6). Such a short photocycle suppresses the normal circadian nocturnal activity and has been useful in uncovering the complementary roles of rod/cone and melanopsin photopigments in negative masking [28]. Typically, activity during the light phase of the day is suppressed, so that the total activity is largely distributed over the three 4 h dark periods. The *rd/rd*, *Opn4^−/−^* and WT mice showed strong negative masking, such that >80% of the daily activity was partitioned into the three dark phases, while both *Opn4^−/−^;rd/rd* and DT-treated *Opn4^Cre/+^;R26^iDTR/+^* mice continued to exhibit strong circadian activity consolidation that was unperturbed by periods of illumination (Figure 6C). They showed equal activity during light and dark phases.

## Discussions

Comprehensive identification and perturbation of genes and circuits are necessary to gain a systems level understanding of circadian regulation of behavior [29]. In the current cellular model of the mammalian circadian system, multiple cell types in the SCN constitute a network of oscillators which is entrained to the ambient light via ocular photoreceptors (reviewed in [30]). The mRGCs have emerged as the predominant RGC sub-type making monosynaptic connection to SCN oscillators.

In this study we generated a mouse line that expresses functional CRE recombinase from the native *melanopsin* locus. We used this mouse line to (a) comprehensively tag mRGC lineage with GFP and to (b) ablate the mRGCs after their normal differentiation. This approach revealed that the *melanopsin* promoter is active in a large number of RGCs, of which ∼40% produce immunologically detectable level of melanopsin protein. Comparable heterogeneity in mRGC morphology (Provencio, personal communication), dendritic arborization [31], light induced calcium responses [11], [32], electrical activity [33] and immunostaining [23] has already been reported. Our approach of transgene tagging from a non-native promoter now allows comprehensive identification of all mRGCs and their classification into two distinct groups based on the level of OPN4 expression. Such differences in melanopsin protein levels along with possible heterogeneity in the relative expression levels of genes defining morphological and functional properties may further contribute to the functional diversity in mRGC population.

The strategy to acutely ablate target cell types in mice with DT-DTR specificity has been successful for many peripheral and intracranial neuronal populations [20], [21], [34], [35], but its efficacy for acute ablation of retina cell types has never been tested. From immunostaining, parallel loss of circadian photoentrainment and other adaptive ocular photoresponses, it is clear that DT can successfully cross the blood-retina barrier in sufficient quantity to trigger ablation of DTR expressing cells. However, we found up to 10% of melanopsin expressing cells escaped cell death due to potential stochastic noise in CRE or DTR expression. It is interesting to note that these surviving cells are unable to generate AOPs. This implies that the SCN and OPN require a strong photic input to reset intrinsic activity. Alternatively, the surviving cells could represent a distinct mRGC population that does not play a role in relaying light information to the SCN or OPN.

Our finding that mRGCs do not overtly disrupt image-forming processes implies a divergence of signals at the level of the retinal ganglion cells. Although the tests employed here did not find any profound loss of image forming function of the retina in acute tests, we cannot rule out the possibility that mRGCs play a subtle direct or indirect role in sculpting the image-forming function of the retina. For instance, in the retina mRGCs may influence function of other RGCs via intercellular coupling [11], they may participate in relaying luminance information to the image processing [18], or by playing an indispensible role in the pupil constriction response, they may reduce direct photodamage to the retina.

We have used the acute cell ablation to understand the cellular bases for light input to the circadian clock. Our results show a central non-redundant role for mRGCs in the relay of photic input to two brain regions that generate adaptive photoresponses. Rod/cone and melanopsin initiated photo signals converge at the level of the mRGCs before relaying to oscillator neurons in the SCN. Similarly, mRGCs appear to serve as the sole functional relay for photic input to the OPN. These results highlight an important cellular design feature of the mammalian circadian system. The mammalian clock, like that of plants, invertebrates and lower vertebrates, uses multiple, mutually compensating photopigments to adapt to the ambient light [36]. However, in other organisms at least some circadian photoreceptors and the clock are cell autonomous and there appear to be multiple molecular nodes for integration of light signal to the molecular clock [25]. The mammalian circadian time keeping system recruits non-cell autonomous multiple photoreceptors, including rod, cones and melanopsin. The complete loss of light entrainment in mRGC ablated mice firmly establishes a unique mammalian-specific cellular network design to integrate light information from these photopigments to the circadian clock. The mRGCs in this design serve as the principal site of signal integration and therefore, have now emerged as a unique cellular target for therapeutic intervention in circadian clock related disorders. The complete loss of non-image forming photoresponses also highlights the role of mRGCs as the principal cellular node where photic information for image-forming and non-image forming responses diverge.

During the course of this study, another manuscript [37] describing specific ablation of mRGCs was published. That study employed direct expression of an attenuated diphtheria toxin from the melanopsin locus, which caused slow degeneration of the mRGCs over several months, as assessed by the loss of a Tau:LacZ transgene expression or melanopsin immunostaining. Both these techniques detect only a subset of mRGCs ([23] and Figure 2). Therefore, the slow degeneration of non-image forming photosensitivity in the study by Guler et al. [37] may have resulted from likely heterogeneity in toxin production from the native promoter, homeostatic synaptic strengthening and/or early developmental compensation. Despite the differences in the pace of cell death, and the approach, both manuscripts arrived at similar conclusion that the mRGCs are the primary node of convergence of melanopsin and rod/cone initiated photoresponses.

## Materials and Methods

### Generation of mice

All animal care and procedures were approved by the Institutional Animal Care and Use Committee of the Genomics Institute of the Novartis Research Foundation and The Salk Institute for Biological Studies. *R26^iDTR/+^* mouse strain was a kind gift of Dr. Ari Waisman [20] and *Z/EG* mouse strain [22] was purchased from Jackson Laboratory. A schematic diagram of the targeting construct for generating the *R26^iDTR/+^* mouse is shown in Figure 1D.

The *Opn4^Cre/+^* mouse was generated by replacing the first seven exons of the mouse *melanopsin* gene with a gene cassette containing Cre (Figure 1D). For generation of the targeting construct, genomic DNA of 129S1SvImJ inbred mouse strain was used as a template and 3.5 kb of genomic sequence immediately upstream of the translation start site and 3.4 kb of sequence 3′ distal to the 7th exon of the of *Opn4* gene were PCR amplified and cloned into two multicloning sites flanking the Cre:IRES:βTau:loxP:Neo:loxP:eYFP cassette. The left arm of the targeting vector also carried a HSV-TK resistance gene. The construct was linearized by NotI digestion and microinjected into an embryonic stem (ES) cell line from 129S mice. ES cell clones with integration of the targeting construct were selected on G418, and 288 antibiotic resistant clones were screened by PCR for homologous recombination of the targeting construct to the *melanopsin* locus. Genomic DNA from seven PCR positive clones was digested with NheI and subjected to Southern blot hybridization with a probe that lies within the right arm. Sequences of the genotyping PCR primers are; wild type (Primer a = CACTTCAGAGACAGCCAGAAGCAGG, Primer b = GACTGACACTGAAGCCTGGCAAACG) and mutant (Primer a and Primer c = CCATTTCCGGTTATTCAACTTGCACC). One clone with appropriately recombined DNA was injected into C57BL/6J blastocysts and introduced into C57BL/6J pseudopregnant females. Chimeric male progeny were bred to C57BL/6J females and the resulting heterozygous agouti coat-colored progeny were mated with C57BL/6J. Heterozygous mice were bred among each other and with *Z/EG* or *Rosa26^iDTR/+^* mice which were also in 129S;C57BL/6 mixed background. Targeted integration of *Cre* leading to loss of melanopsin protein in *Opn4^Cre/Cre^* mice was verified by lack of anti-OPN4 immunostaining (data not shown). *Opn4^Cre/+^;Z/EG* mice were used for assessment of CRE function and fluorescent tagging of mRGCs in the retina. Littermate *Opn4^Cre/+^;R26^iDTR/+^* and WT mice were used in all subsequent experiments for acute ablation of mRGCs.

The *Opn4^−/−^* mice used in this study were generated in 129S background and characterized earlier [15]. These mice were bred to a line of C57BL/6 carrying the *Pde6b^rd/rd^* mutation. Progeny from this breeding, which were also in a 129S;C57BL/6 background were genotyped and mice of *Opn4^−/−^*, *rd/rd* and *Opn4^−/−^;rd/rd* genotypes were used for behavioral studies.

### DT injection

DT (D0564, Sigma-Aldrich, St. Louis, MO) was dissolved in sterile PBS (1 mg/ml) and stored at −80°C till use. Freshly thawed DT stock solution was diluted in sterile PBS and injected intraperitoneally (50 μg/kg body weight) [21] to 8–12 weeks old *Opn4^Cre/+^;R26^iDTR/+^* and WT littermate mice. The dose was repeated once or twice at 3 d interval.

### Retina staining

For flat mount, adult mice were sacrificed, the eyes quickly removed and placed into aerated Ames medium (Sigma-Aldrich, St. Louis, MO). After removal of the corneas and lenses, eyecups were fixed in 4% paraformaldehyde for 15 min. Following three washes in PBS, retinas were dissected from eyecups, stretched onto filter paper, and processed in 24-well plates. The retinas were incubated in a blocking solution (0.3% Triton X-100, 5% normal donkey serum, and 0.5% glycine in PBS) for 1 h at room temperature. After three washes in PBS, the retinas were incubated in a 1:5,000 dilution of rabbit anti-OPN4 antiserum (against a peptide consisting of the 15 N-terminal amino acids of mouse melanopsin [38]) or in 1:500 dilution of rabbit anti-GFP antibody (Cat# A11122, Invitrogen, Carlsbad, CA) in the blocking solution for overnight at 4°C and rinsed with PBS. Melanopsin immunoreactivity was visualized with Cy3-conjugated donkey anti-rabbit IgG (1:500, Cat# 711-165-152, Jackson ImmunoResearch Laboratories, West Grove, PA) or FITC-conjugated anti-rabbit IgG (1:20, Cat# 401314, Calbiochem, San Diego, CA) in blocking solution. Finally, retinas were washed with PBS and mounted with PermaFluor (Cat# IM0752, Beckman Coulter, Fullerton, CA). Fluorescent images were captured using an Olympus Fluoview500 confocal microscope or Leica TCS SP2 AOBS confocal microscope. For Hematoxylin and Eosin (H&E) staining, eyes were removed and eyecups were fixed in 4% paraformaldehyde for 2 h, washed three times with PBS, and embedded in paraffin. Five micron thick paraffin sections were used for H&E staining. Stained slides were visualized under a Leica microscope.

### Anterograde tracing with cholera toxin

Mice were anesthetized with ketamine (70 mg/kg) and xylazine (10 mg/kg) and one drop each of 1% tropicamide and 0.5% proparacaine (Bausch & Lomb, Tampa, FL) were applied to their eyes. An incision was made with a 30 gauge needle below the limbus region and 1 μl of 1% Cholera Toxin-B subunit (CTB) conjugated to Alexa Fluor 488 (Cat# C34775, Invitrogen, CA) was injected into the vitreous. After 48 h, the mice were anesthetized with ketamine (70 mg/kg) and xylazine (10 mg/kg), perfused with 4% paraformaldehyde in 1×PBS. The brain was extracted, further fixed at 4°C overnight, embedded in OCT and 40 μm thick sections cut in a microtome were imaged using an Olympus Fluoview500 confocal microscope.

### ERG

Mice were dark adapted for 1 h, anesthetized with ketamine (70 mg/kg) and xylazine (10 mg/kg). The eyes were applied with 1% tropicamide, 0.5% proparacaine and Genteal lubricant eye gel (CIBA vision, Duluth, GA, USA). Electrodes were connected under dim red light. ERGs were recorded from both eyes with an ERG system (Model LE-2000, Tomey, Nagoya, Japan). White LED light embedded in contact lens recording electrodes were used as light sources. ERG recordings were done following the standards of the International Society for Clinical Electrophysiology of Vision (ISCEV; http://www.iscev.org/standards/erg1999.html). After an additional 5 min of dark adaptation, rod response was measured by averaging 4 dim light flashes of 80 cd.m^−2^×0.12 msec at >2 sec interval. Then mice were further dark adapted for 3 min and maximal combined (rod and cone) response and oscillatory potential were determined from the average of responses to four flashes of 6,000 cd.m^−2^×0.5 msec at >10 sec inter-stimulus interval. Finally, after 10min light adaptation (25 cd.m^−2^), average single-flash cone response was determined from 4 flashes of 6,000 cd.m^−2^×0.5 msec at >15 sec interval.

### Visual cliff test

The visual cliff test was assessed as described in [39]. Half of a 31 cm length×45 cm width×50 cm tall clear acrylic box had a black and white checkerboard pattern on the top (the “safe” side) and the other side had a clear top (the “unsafe” side/cliff side). The unsafe side had a checkerboard pattern on the bottom to give an illusion of added depth. Black paper was placed on the inside of the walls to reduce reflections. A block of 31 cm length×3.5 cm width×2.5 cm height was placed at the center of the box to separate these two sides. The mouse was placed on the block and allowed to make a spontaneous step down between the safe side and the unsafe side. Data was measured as positive when the mouse chose to step down onto the horizontal checkered surface (the safe side) and as negative onto the clear side (the unsafe side). Each mouse was tested for 10 consecutive trials. Fifty percent positive indicated that the mouse moved off the ridge onto the two sides with random chance.

### PLR

PLR was assessed as described in [40] with some modification. Mice were dark adapted for at least an hour prior to PLR assessment. Unanaesthetized mice were hand restrained with the left eye apposed to one of the outlets of an integrating sphere. The pupil of right eye was video monitored under infrared light with a consumer grade digital video camera. An additional +2 diaptor lens and an infrared filter were fitted to the light path of the camera to improve the image quality. After collecting images of dark adapted pupils, the contralateral eye was exposed to light from the integrating sphere. Light from a 300 Watt Xenon Arc lamp light source (Sutter Instrument, Novato, CA, USA) was filtered, collimated and delivered to the integrating sphere through a liquid light guide. An inline 470 nm filter, a filter wheel with a series of neutral density filters, and a Lambda 10-3 optical filter changer with SmartShutter™ were used to control the spectral quality, intensity and duration of light. Light intensity was measured with a Melles Griot power meter. Video images of pupil constriction were analyzed by ViewPoint EyeTracker™ software (Arrington Research Inc., Scottsdale, AZ). Dark adapted pupil area prior to light pulse and pupil area after 30 sec of light were measured and pupil constriction was defined as 1-(dark adapted pupil width)^2/(pupil width after 30 sec of light)^2. The squares come in to account for the calculation of pupil area. Pupil area constriction was plotted against the log of irradiance. Data was fitted to a sigmoidal dose response curve with the lower asymptote fixed to y = 0 using Origin lab 8 software.

For tracking the gradual changes in PLR response following DT injection, pupil area constrictions over 8 days were normalized to maximum constriction taken as 100%.

### Locomotor activity measurement

Daily locomotor activity of mice individually housed in wheel running cages was measured as described in [41]. Typically, 6–10 week old mice were individually housed in wheel running cages placed inside light tight boxes with independent illumination. During the light phase, the mice received ∼150 lux of white light from fluorescent light source. Wheel running activity in 1 min bins was continuously collected and later analyzed by Clocklab software (Actimetrics, Evanston, IL, USA). All routine animal husbandry practices during the dark phase were performed under dim red light.
