# Correction: Inducible Ablation of Melanopsin-Expressing Retinal Ganglion Cells Reveals Their Central Role in Non-Image Forming Visual Responses

**DOI:** 10.1371/annotation/c02106ba-b00b-4416-9834-cf0f3ba49a37

**Published:** 2008-07-31

**Authors:** Megumi Hatori, Hiep Le, Christopher Vollmers, Sheena Racheal Keding, Nobushige Tanaka, Thorsten Buch, Ari Waisman, Christian Schmedt, Timothy Jegla, Satchidananda Panda

Drs. Thorsten Buch, and Ari Waisman were not included in the Author byline. They should be listed as the sixth and seventh authors, respectively. Please view the full author list and affiliations here:

Megumi Hatori^1^, Hiep Le^1^, Christopher Vollmers^1^, Sheena Racheal Keding^1^, Nobushige Tanaka^1¤^, Thorsten Buch^2^, Ari Waisman^3^, Christian Schmedt^4^, Timothy Jegla^5^, Satchidananda Panda^1*^

1 The Salk Institute for Biological Studies, La Jolla, California, United States of America

2 Institute of Experimental Immunology, University of Zurich, Zurich, Switzerland

3 I.Medizinische Klinik und Poliklinik, Johannes Gutenberg-Universität Mainz, Mainz, Germany

4 Genomics Institute of Novartis Research Foundation, San Diego, California, United States of America

5 Department of Cell Biology and Institute for Childhood and Neglected Diseases, The Scripps Research Institute, La Jolla, California, United States of America 

* To whom correspondence should be addressed. E-mail: satchin@salk.edu 

¤ Current address: Kyorin University, Tokyo, Japan 

