# Correction: Inducible Ablation of Melanopsin-Expressing Retinal Ganglion Cells Reveals Their Central Role in Non-Image Forming Visual Responses

**DOI:** 10.1371/annotation/16f913dd-c33b-419f-9555-c788c80c189f

**Published:** 2008-07-31

**Authors:** Megumi Hatori, Hiep Le, Christopher Vollmers, Sheena Racheal Keding, Nobushige Tanaka, Thorsten Buch, Ari Waisman, Christian Schmedt, Timothy Jegla, Satchidananda Panda

Because two authors were added to the author byline, the Author Contributions do not correctly reflect the new authorship. The "Contributed reagents/materials/analysis tools" section should now read: CS TJ TB AW.

